# Role of ghrelin in the pancreatic exocrine secretion via mitogen-activated protein kinase signaling in rats

**DOI:** 10.1186/s40781-017-0141-9

**Published:** 2017-07-24

**Authors:** Kyung-Hoon Lee, Jae-Sung Lee, Tao Wang, Jin-Ju Oh, Sanggun Roh, Hong-Gu Lee

**Affiliations:** 1 Department of Animal Science and Technology, College of Animal Bioscience and Technology, Konkuk University, Seoul, 05029 South Korea; 2 Research Department, Korea Industrial Co., Ltd., Pusan, 46978 South Korea; 3 Team of Educational Program for Specialists in Global Animal Science, Brain Korea 21 Plus Project, Konkuk University, Seoul, 05029 South Korea; 4 College of Animal Science and Technology, Jilin Agricultural University, Changchun, 130118 China; 5 Natural Product Clinical Research Center, Clinical Research Center, Pusan National University School of Medicine, Busan, 49241 South Korea; 6 Laboratory of Animal Physiology, Graduate School of Agricultural Science, Tohoku University, Sendai, 981-8555 Japan

**Keywords:** α-Amylase activity, Cholecystokinin, Ghrelin, Pancreatic exocrine, Sprague-Dawley rats

## Abstract

**Background:**

This study was performed to investigate the impact of exogenous ghrelin on the pancreatic α-amylase outputs and responses of pancreatic proteins to ghrelin that may relate to pancreatic exocrine.

**Methods:**

Sprague-Dawley male rats (9 weeks old, 300 ± 10 g) were injected with ghrelin via intraperitoneal (i.p.) infusion at dosage of 0, 0.1, 1.0 and 10.0 μg/kg body weight (BW), respectively. The plasma ghrelin and cholecystokinin (CCK) level were determined using enzyme immunoassay kit; the mRNA expression of ghrelin receptor (GHSR-1α) and growth hormone (GH) receptor were assessed by reverse transcription PCR; the expressions of pancreatic α-amylase activity, extracellular-signal-regulated kinases (ERK), phosphorylated extracellular-signal-regulated kinases (pERK) and c-Jun N-terminal kinase (JNK) were evaluated by western blotting; moreover the responses of pancreatic proteins to ghrelin were analyzed using the two-dimensional gel electrophoresis system.

**Results:**

The exogenous ghrelin (1.0 and 10.0 μg/kg BW) elevated the level of plasma ghrelin (*p* < 0.05), and suppressed the expression of pancreatic α-amylase at a dose of 10.0 μg/kg BW (*p* < 0.05). No difference in the level of plasma CCK was observed, even though rats were exposed to any dose of exogenous ghrelin. In addition, a combination of western blot and proteomic analysis revealed exogenous ghrelin (10.0 μg/kg BW) induced increasing the JNK and ERK expressions (*p* < 0.05) and four proteins such as Destrin, Anionic trypsin-1, Trypsinogen, and especially eukaryotic translation initiation factor 3 in rat pancreas.

**Conclusions:**

Taken together, exogenous ghrelin by i.p. infusion plays a role in the pancreatic exocrine secretion via mitogen-activated protein kinase signaling pathway.

## Background

Ghrelin is a peptide hormone that has been isolated from the stomach and plays important roles in the release of growth hormone (GH), appetite stimulation and various physiological activities [1, 2]. Ghrelin receptor (GHSR-1α) detected in endocrine cells of the stomach or/and in pancreatic cells shows a number of actions in the intestines and in the pancreas. Intravenous administration of ghrelin in rats reduced the secretion of pancreatic enzymes stimulated by cholecystokinin (CCK) and the suppressible response of the pancreas to ghrelin is indirect and may be exerted at the level of intra-pancreatic neurons [3]. On the other hand, central administration of ghrelin stimulated pancreatic exocrine secretion in conscious rats [4]. In addition, the ghrelin acts on dorsal vagal complex to stimulate pancreatic protein output [5]. Especially, ruminants have a limited ability for intestinal starch digestion of pancreatic α-amylase [6]. Previous studies [7, 8] suggested that the supply of gastrointestinal hormones also can improve starch digestion in the bovine small intestine, showing that α-amylase secretion from the pancreas for starch digestion is involved in gastrointestinal hormones, CCK and ghrelin. Such CCK and secretin were widely revealed that these things increased pancreatic α-amylase secretion. However, the results of previous studies concerning the influence of ghrelin on pancreatic exocrine function are still controversial.

We therefore performed this study to determine the impacts of exogenous ghrelin, given intraperitoneal (i.p.) on plasma ghrelin, CCK level and pancreatic α-amylase output in the Sprague-Dawley (SD) rats. In addition, the responses of pancreatic proteins to exogenous ghrelin were investigated using the two-dimensional gel electrophoresis (2-DE) system.

## Methods

### Animals and management

Nine-week-old SD male rats (Samtako Bio Korea Co. Ltd., Osan-si, Gyeonggi-Do, South Korea) with average body weights (BW) of 300 ± 10 g were used for all experiments. Animals were housed at one cage per animal on a 12/12 h light/dark cycle (lights on at 08:00 h) and given access to food and water ad libitum.

### Sampling procedures

Exogenous ghrelin (Acylated form; Bachem Americas, Inc., Torrance, CA, USA) was injected in rats by i.p. infusion at doses of 0, 0.1, 1.0 and 10.0 μg/kg BW, respectively. One hour after the injection, each blood sample (1 mL) was collected from rat caudal vein once into heparined-tube and immediately centrifuged (3000 rpm, 15 min) to obtain the plasma in all group. Aliquots of plasma were stored at −80 °C till analyzed. Rats were then anesthetized via intramuscular injection of zoletil (Virbac, Carros, France) at a dose of 15.0 mg/300 g BW and each pancreas, liver and pituitary tissue of rats were aseptically collected. All experimental procedures were in accordance with the Animal Care and Use Committee of Pusan National University (PNU-2012-0056).

### Determination of plasma ghrelin and CCK level

Rat ghrelin peptide was obtained from Bachem Americas, Inc. (Torrance, CA, USA). Rat plasma ghrelin and CCK enzyme immunoassay kit were purchased from Phoenix Pharmaceuticals Inc. (Burlingame, CA, USA). All the measurements were done following the manufacturers’ instruction.

### Ribonucleic acid (RNA) extraction and reverse transcription polymerase chain reaction (RT-PCR)

RT-PCR was performed to assess the messenger RNA (mRNA) expression of ghrelin receptor (GHSR-1α) and GH receptor. Total RNA was individually extracted from the pancreas, liver and pituitary of rat given ghrelin (10.0 μg/kg BW) using TRIzol Reagent (Invitrogen, Carlsbad, CA, USA). First-strand complementary deoxyribonucleic acid was synthesized from 5 g of RNA and 1 g oligo (dT) primer with 250 units M-MLV reverse transcriptase (Invitrogen, Carlsbad, CA, USA) and then subjected to PCR amplification using gene specific primers. The PCR specific primers used in this study were 5′-GAGATCGCTCAGATCAGCCAGATCAGCCAGTAC-3′ (sense) and 5′-TAATCCCCAAACTGAGGTTCTGC-3′ (anti-sense) for GHSR-1α, 5′-GAAAGAATGCCCTGATTATGTC-3′ (sense) and 5′-TCATACTCCAGAATTATCCATCC-3′ (anti-sense) for GH receptor. The RT-PCR conditions were 32 cycles of denaturation at 94 °C for 30 s, annealing at 59.5 °C for 30 s and elongating at 72 °C for 30 s. The PCR products were resolved on a 1.5% agarose gel by electrophoresis.

### Western blot analysis

Pancreatic tissues of rats given exogenous ghrelin (10.0 μg/kg BW) were homogenized and suspended in lysis buffer containing 50 mM Tris-hydrochloric acid (HCl) (pH 8.0), 1% sodium dodecyl sulfate, 0.25% sodium deoxycholate, 1 mM ethylenediaminetetra acetic acid, and 1 X protease inhibitor (GE Healthcare, Piscataway, NJ, USA). Extracts were incubated for 30 min on ice and stirred every 5 min, after which the samples were centrifuged at 14,000 rpm for 30 min at 4 °C. The protein concentrations were determined by a BCA Protein Assay (Pierce, Rockford, IL, USA). For western blot of pancreatic α-amylase, extracellular-signal-regulated kinases (ERK), phosphorylated extracellular-signal-regulated kinases (pERK) and c-Jun N-terminal kinase (JNK), 30 μg protein was separated by 12.5% (*w*/*v*) sodium dodecyl sulfate polyacrylamide gel electrophoresis (SDS-PAGE), after which the samples were blotted onto nitrocellulose membranes. The membranes were blocked overnight at 4 °C in TBST (20 mM Tris-HCl, pH 7.6), 137 mM sodium chloride, 0.01% Tween 20) containing 5% skim milk and then incubated for 3 h at 4 °C with the following primary antibodies: Rabbit polyclonal to pancreatic α-amylase (1:16,000, 54 kDa), rabbit polyclonal to ERK (sc-94, 1:1000, 42/44 kDa), rabbit polyclonal to SAPK/JNK (#9252, 1:1000, 46/54 kDa) (Cell signaling Technology, Danvers, MA, USA). The samples were then washed three times with 1X TBST, after which they were incubated with the appropriate secondary antibody for 3 h at 4 °C: Goat polyclonal to rabbit IgG-HRP (ab6721, 1: 10,000, Abcam, Cambridge, MA, USA). The proteins on the membrane were visualized using and enhanced chemiluminescence system plus detection kit (GE Healthcare, Piscataway, NJ, USA), after which they were exposed to X-ray film (Fujifilm Co., Tokyo, Japan) for 1-3 min. The films were then scanned and the bands were quantified using the Image J 1.43 software (http://rsb.info.nih.gov/ij/download.html).

### 2-DE analysis

2-DE was performed with proteins of pancreatic tissue of rats given exogenous ghrelin (10.0 μg/kg BW). Briefly, protein samples were diluted into isoelectric focusing (IEF) buffer containing 6 M urea, 2 M thiourea, 1% 3-[(3-cholamidopropyl) dimethylammonium]-1-propanesulfonate, 0.002% bromophenol blue, 0.5% pharmalyte (pH 3-10NL) and 65 mM dithiothreitol (DTT). Then 100 μg protein samples of control or treatment were loaded on Immobiline DryStrip gels (pH 3-10NL, 18-cm; GE Healthcare, Piscataway, NJ, USA) for rehydration for 12 h at 20 °C. The IEF procedures were performed using an IEF electrophoresis unit (GE Healthcare, Piscataway, NJ, USA) following the manufacturer’s protocols. The following voltage program was used after the 12 h rehydration: linear ramp from 500 to 1000 V over 2 h, then a constant voltage of 8000 V for 7 h to give a total of 56,000 V h. After focusing, gel strips were equilibrated in a solution containing 50 mM Tris-HCl (pH 8.8), 6 M urea, 2% sodium dodecyl sulfate, 30% glycerol, 0.002% (*w*/*v*) bromophenol blue and DTT for 15 min, followed by incubation in the same solution but replacing DTT with 135 mM iodoacetamide for another 15 min. After that the equilibrated strips were inserted into SDS-PAGE gels (18-cm, 12%). SDS-PAGE was performed using an Ettan DALT 2-D gel system (GE Healthcare, Piscataway, NJ, USA). Upon completion, gels were stained using a PluseOne™ Silver Staining Kit (GE Healthcare, Piscataway, NJ, USA). The silver-stained gels were scanned using an Umax scanner (Power Look 2100XL, UMAX Technologies Inc., Dallas, TX, USA). Scanned gel images were processed by Proteomweaver™ 2-D Analysis Software (Definiens AG, Munich, Germany).

### Statistical analysis

All values are presented as means ± standard error of the mean (SEM). The group mean values were compared with an independent sample *t*-test (SPSS 14.0, Chicago, IL, USA). A probability less than 0.05 was considered to be statistically significant.

## Results

**Fig. 1:**
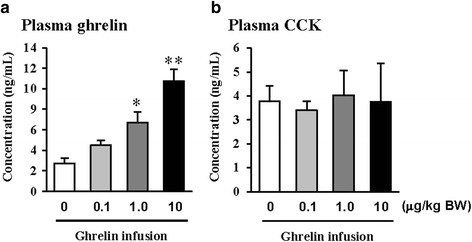
Concentration of plasma ghrelin (**a**) and cholecystokinin (CCK) (**b**) in rat exposure to exogenous ghrelin (0, 0.1, 1.0 and 10.0 μg/kg BW). The values are expressed as the average with SEM (*n* = 4). Asterisk shows significant difference with the ghrelin-untreated group (*p < 0.05, **p < 0.01)

**Fig. 2:**
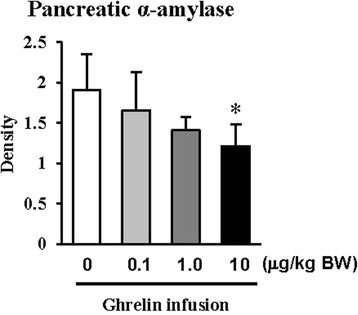
Western blot analysis of α-amylase expressions in tissues of pancreas of rat exposure to exogenous ghrelin (0, 0.1, 1.0 and 10.0 μg/kg BW). The values are expressed as the average with SEM (*n* = 4). Asterisk shows significant difference with the ghrelin-untreated group (*p < 0.05)

**Fig. 3:**
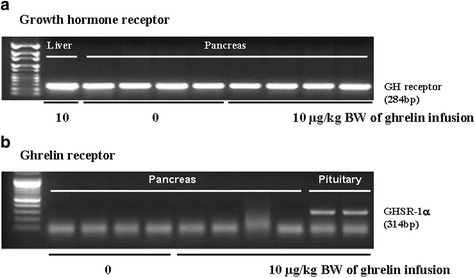
mRNA expressions of pancreatic ghrelin receptor (GHSR-1α) and growth hormone (GH) receptor in tissues of pancreas, liver, and pituitary of rat exposure to exogenous ghrelin (0 and 10.0 μg/kg BW) using RT-PCR

**Fig. 4:**
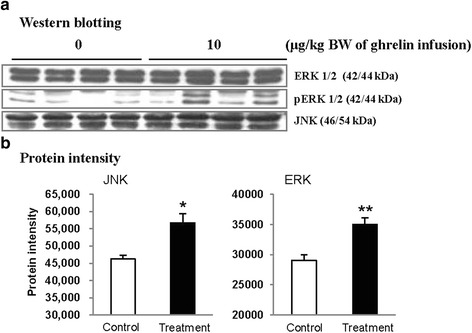
Level of pancreatic extracellular-signal-regulated kinases (ERK), phosphorylated extracellular-signal-regulated kinases (pERK) and c-Jun N-terminal kinase (JNK) expression by using western blotting. Protein sample from pancreatic tissue of rats treated with ghrelin (10.0 μg/kg BW) was analyzed by western blot (**a**) and their bands were determined by pixel intensity analysis using NIH Image J (**b**). The values are expressed as the average with SEM (*n* = 4). Asterisk shows significant difference with the ghrelin-untreated group (*p < 0.05, **p < 0.01)

**Fig. 5:**
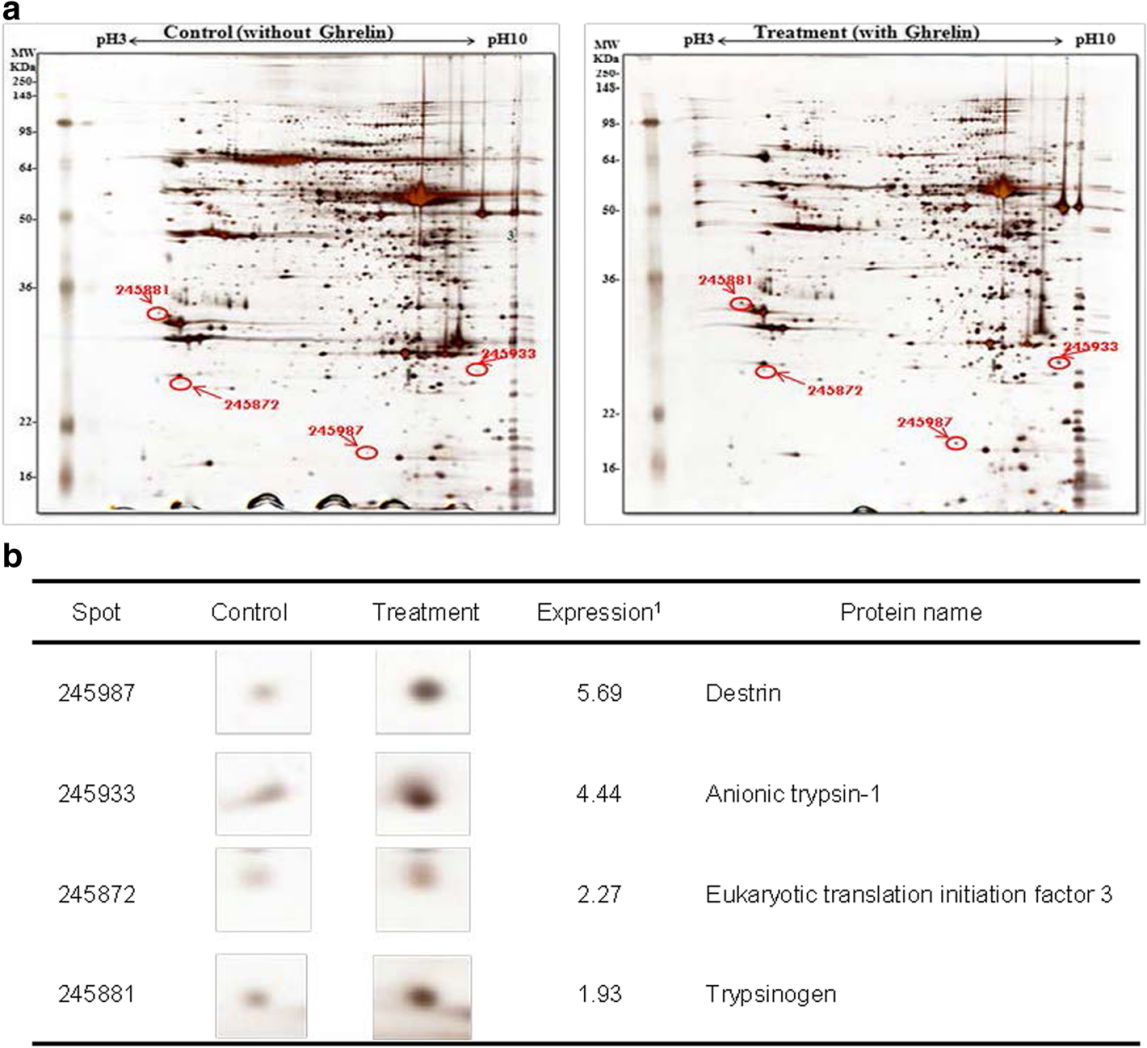
Identification of differentially altered protein spots in rat pancreas by ESI/Q-TOF MS. Representative silver-stained 2-DE images of the normal rat pancreas (*left*) and 10.0 μg/kg BW ghrelin-infused rat pancreas (*right*) (**a**). The spots that differentially expressed proteins in rat pancreas were identified by ESI/Q-TOF MS (**b**). ^1)^ The values of the protein expression were presented as the ghrelin-treated group against ghrelin-untreated group

In this study, the i.p. infusion of ghrelin (0, 0.1, 1.0 or 10.0 μg/kg BW) significantly elevated plasma concentrations of ghrelin (*p* < 0.05) (Fig. 1a). Nevertheless increasing plasma ghrelin, no significant differences in the plasma CCK level were observed (Fig. 1b). Western blot analysis revealed that increasing plasma ghrelin decreased pancreatic α-amylase expression (Fig. 2). Based on our observation, the ghrelin receptor (GHSR-1α) does not exist (Fig. 3B); however, we found that the GH receptor mRNA expression was observed in the pancreatic tissue of rats given exogenous ghrelin (10.0 μg/kg BW, Fig. 3A). We found that the expression of JNK and ERK in ghrelin-treated group (10.0 μg/kg BW) was higher than that in untreated group (Fig. 4). Moreover, four spots (Destrin, Anionic trypsin-1, Trypsinogen and Eukaryotic translation initiation factor 3) ranging from 6 to 200 kDa were detected by 2-DE, and differently expressed in the pancreatic protein map (Fig. 5).

## Discussion

In the previous study, we showed that increasing plasma CCK level did not significantly increase pancreatic α-amylase expression [9]. However, it differed from those of other previous studies [4, 5, 10, 11]. We therefore hypothesized that the mechanism related to pancreatic exocrine secretion may have down-regulating factor by exogenous ghrelin. We believed that GH might be one of these factors. Studies by Anderson and Hellman et al. [12, 13] reported that GH decreases insulin secretion via mitogen-activated protein (MAP) kinase signaling and has a paracrine effect on endocrine secretion. Thus, we investigated the levels of plasma ghrelin and CCK by i.p. infusion with exogenous ghrelin (0, 0.1, 1.0 and 10.0 μg/kg BW). The i.p. infusion of ghrelin at the dose of 1.0 and 10.0 μg/kg BW significantly increased plasma concentrations of ghrelin (*p* < 0.05) whereas no significant differences in the plasma CCK level was observed (Fig. 1a, b). The western blot analysis of pancreatic α-amylase activity (Fig. 2) suspected that ghrelin may regulate exocrine secretion through other signals; direct or indirect actions not releasing CCK. Thus, we assumed the signal pathway related to pancreatic α-amylase secretion either ghrelin’s direct action or other factors. To further elucidate the mechanism related to pancreatic α-amylase secretion, ghrelin receptor (GHSR-1α) and GH receptor mRNA expression were examined using RT-PCR (Fig. 3). In addition, the results indicated ghrelin may indirectly influence the pancreatic α-amylase secretion via releasing GH.

Based on this finding, we hypothesized that ghrelin may decrease pancreatic α-amylase secretion via the release of GH. Therefore, we determined whether GH releases affected pancreatic α-amylase secretion. Studies reported that MAP kinase signaling concerned with GH affects the pancreatic endocrine secretion and paracrine effect to exocrine secretion [12, 13]. Thus, we confirmed JNK and ERK expressions, one of MAP kinase signal factors. As shown in the Fig. 4, the expression of JNK and ERK in ghrelin-treated group (10.0 μg/kg BW) was higher than that in untreated group (0 μg/kg BW) (*p* < 0.05). It has been reported that expression of JNK and ERK is stimulated by GH [14]. We therefore speculated that these factors were expressed by ghrelin stimulating the release of GH. Studies showed that MAP kinase was found to exert anti-insulin effect by inducing the release of GH. This anti-insulin activity inhibits pancreatic exocrine secretion [12, 14]. Synthetically, we suggested that increasing expression of factors in the MAP kinase signaling by GH induced the decrease in the pancreatic exocrine secretion. The response of pancreatic proteins to exogenous ghrelin (10.0 μg/kg BW) by i.p. infusion was analyzed by 2-DE in order to get a better understanding of the mechanisms involved in the ghrelin exocrine secretion through GH signal (Fig. 5). Four spots were up-regulated by exogenous ghrelin infusion compared to untreated group (0 μg/kg BW). Among these spots, proteins of eukaryotic translation initiation factor 5A-1 (eIF5A) and destrin were of particular interest due to that destrin is involved in decreasing insulin secretion [14, 15]. Our study found that the destrin expression was elevated by releasing GH response to exogenous ghrelin, indicating that exogenous ghrelin by i.p. infusion could be acted as a modulator for insulin secretion. eIF5A is involved in biosynthesis of pancreatic enzyme by increasing plasma CCK level [16]. In contrast, our study showed exogenous ghrelin had no influence on plasma CCK level (Fig. 1b). Considering our present reports, ghrelin may have effect on pancreatic exocrine secretion via other factors in releasing CCK. When the effects of exogenous ghrelin by i.p. infusion on pancreatic exocrine secretion was done artificially, we have to consider other factors like GH related to insulin synthesis as involving in the CCK secretion and activation of cholinergic vago-vagal enteropancreatic reflex.

## Conclusion

The result suggested exogenous ghrelin by i.p. infusion in rats modulates the secretion of pancreatic exocrine via MAP kinase signaling, such as JNK and ERK proteins. However, the proteins related to functional study mechanism were still uncompleted. Thus, some following functional study of proteins will be investigated in the near future.

## Abbreviations

%: Percent
°C: Celsius
2-DE: Two-dimensional gel electrophoresis
BW: Body weight
CCK: Cholecystokinin
eIF5A: Eukaryotic translation initiation factor 5A-1
ERK: Extracellular-signal-regulated kinases
g: Gram
GH: Growth hormone
H: Hour
i.p.: Intraperitoneal
JNK: c-Jun N-terminal kinase
Kg: Kilogram
M: Molar
MAP: Mitogen-activated protein
Mg: Milligram
Min: Minute
mM: Millimolar
mRNA: messenger RNA
PCR: Polymerase chain reaction
pERK: Phosphorylated extracellular-signal-regulated kinases
rpm: Revolutions per minute
RT: Reverse transcription
S: Second
SD: Sprague-Dawley
SDS-PAGE: Sodium dodecyl sulfate polyacrylamide gel electrophoresis
SEM: Standard error of the mean
Μg: Microgram
